# Apocrine Ductal Carcinoma *in situ* Ex Pleomorphic Adenoma of the Breast: A Rare Case Report

**DOI:** 10.70352/scrj.cr.25-0771

**Published:** 2026-03-20

**Authors:** Tadahiro Isono, Ryohei Koreyasu, Konomi Sakyo, Ryosuke Kishi, Takahiro Watanabe, Takeshi Ueda, Masashi Nozawa, Kazuyasu Kamimura, Mitsuhiro Tachibana, Hidetoshi Wada

**Affiliations:** 1 Department of Surgery, Shimada General Medical Center, Shimada, Shizuoka, Japan; 2 Diagnostic Pathology, Shimada General Medical Center, Shimada, Shizuoka, Japan

**Keywords:** breast cancer, ductal carcinoma *in situ*, carcinoma ex pleomorphic adenoma, apocrine, case report

## Abstract

**INTRODUCTION:**

Pleomorphic adenoma (PA) of the breast is an extremely rare benign tumor, and malignant transformation within PA is exceptional. Only a few cases have been reported, and none have described apocrine ductal carcinoma *in situ* (DCIS) arising in PA. We report a case of apocrine-type DCIS considered to have developed from mammary PA.

**CASE PRESENTATION:**

A 75-year-old woman presented with a 2.0-cm palpable mass in the left breast with nipple inversion. Mammography showed a well-circumscribed 24-mm mass and an adjacent 11-mm lesion with irregular margins near the nipple. MRI demonstrated a cystic lesion with an enhancing mass on the nipple side and nipple retraction toward the lesion. Cytology of cyst fluid revealed no malignant cells and was consistent with a benign hemorrhagic cyst. However, core needle biopsy of the adjacent lesion identified apocrine-type DCIS. Systemic evaluation showed no metastases, and the clinical diagnosis was cTisN0M0, cStage 0 breast cancer. Because nipple involvement could not be excluded, nipple-sparing surgery was not recommended, and the patient underwent mastectomy. Histopathology revealed a well-defined, encapsulated PA containing an intraductal apocrine carcinoma component. The carcinoma cells showed abundant eosinophilic cytoplasm, high nuclear grade, and occasional mitoses without stromal invasion. Immunohistochemically, tumor cells were androgen receptor and Forkhead box protein A1-positive and estrogen- and progesterone receptor-negative, with a Ki-67 index of approximately 30%. The lesion was diagnosed as apocrine-type DCIS ex PA, with ductal extension toward the nipple corresponding to the clinical nipple retraction.

**CONCLUSIONS:**

We describe the first reported case of apocrine-type DCIS arising in breast PA. Awareness of this rare entity is essential to avoid misdiagnosis and to clarify its clinicopathological characteristics.

## Abbreviations

AR
: androgen receptor
CEPA
: carcinoma ex pleomorphic adenoma
DCIS
: ductal carcinoma *in situ*
ER
: estrogen receptor
FOXA1
: forkhead box protein A1
PA
: pleomorphic adenoma
PgR
: progesterone receptor

## INTRODUCTION

Pleomorphic adenoma (PA) is a benign mixed tumor most commonly observed in the salivary glands. Although the mammary glands share histological similarities with the salivary glands, PA arising in the breast is extremely rare and typically occurs in the subareolar region. It has been suggested that PA in the breast may originate from an intraductal papilloma. Malignant transformation of PA, known as carcinoma ex PA (CEPA), has been reported in salivary glands and other sites; however, CEPA of the breast is exceedingly uncommon, with only 3 prior reports describing 6 cases.

Here, we report the seventh documented case of CEPA in the breast and the first case of apocrine ductal carcinoma *in situ* (DCIS) arising from PA. In this patient, a PA was located near the nipple, from which DCIS developed and extended within the ducts toward the nipple. Multiple cysts of varying sizes were observed in the peripheral breast tissue. The PA and DCIS likely caused ductal obstruction, leading to cyst formation, while the tumor and associated DCIS contributed to nipple retraction.

This case highlights the potential for malignant transformation in PA of the breast and underscores the importance of careful histopathological evaluation when subareolar masses are identified. Recognition of this rare entity is crucial to avoid misdiagnosis and to guide appropriate surgical management. Reporting such cases contributes to understanding the clinicopathological characteristics and biological behavior of PA and its breast malignancies.

## CASE PRESENTATION

**Fig. 1:**
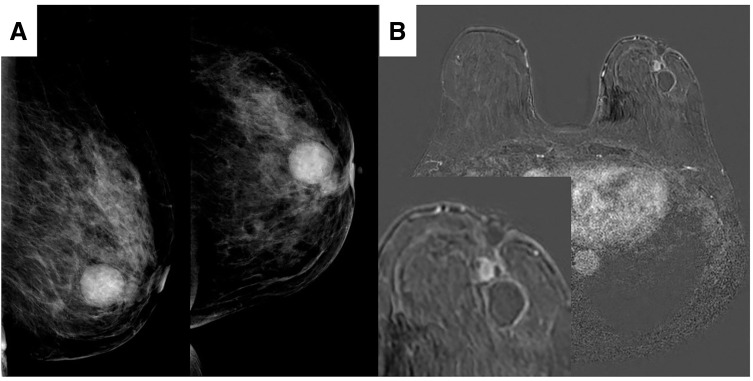
Imaging findings. (**A**) Mammography shows a well-circumscribed, smooth mass measuring 24 mm in the left breast, along with an additional 11-mm mass with microlobulated and irregular margins located on the nipple side adjacent to the cystic lesion. (**B**) MRI of the breast revealed a 25-mm cystic lesion with an adjacent 10-mm enhancing mass on the nipple side; the nipple was retracted toward the side of the mass.

The patient was a 75-year-old woman who noticed a 2.0-cm breast mass near the left nipple. She was suspected of having breast cancer at a local clinic and was referred to our hospital for further examination and treatment. She had been receiving medication for hypertension and hyperlipidemia since her 50s. There was no family history of breast cancer or other malignancies. The tumor was palpable in the E region of the left breast, measuring 2.0 cm in diameter. It was freely mobile, with no skin changes observed, although nipple inversion was present. On the craniocaudal view of the mammogram, a well-circumscribed, smooth mass measuring 24 mm was observed in the left breast, along with an additional 11-mm mass with microlobulated and irregular margins located on the nipple side (**Fig. 1A**). Breast MRI demonstrated a cystic lesion with an adjacent enhancing mass on the nipple side, with retraction of the nipple toward the lesion (**Fig. 1B**). The enhancing mass was a well-defined mass with smooth margins measuring 9 mm in diameter. It showed signal intensity comparable to that of the background breast parenchyma on fat-suppressed T1-weighted images and low signal intensity on fat-suppressed T2-weighted images. Diffusion-weighted imaging demonstrated high signal intensity with corresponding low apparent diffusion coefficient values. In the dynamic contrast-enhanced study, enhancement appeared nearly simultaneously with the background breast parenchyma during the ultrafast phase. In the early post-contrast phase, the lesion exhibited homogeneous enhancement, and the time–intensity curve demonstrated a fast plateau pattern. Unfortunately, the preoperative ultrasound images were lost due to an error in the image storage procedure. Cytological examination of the cystic fluid revealed no apparent mammary epithelial cells. The background showed hemorrhage and macrophages containing hemosiderin, suggesting a benign hemorrhagic cystic lesion. Core needle biopsy of the tumor confirmed it as apocrine-type DCIS. Systemic evaluation, including CT, showed no evidence of distant metastasis, and the patient was diagnosed with cTisN0M0, corresponding to clinical stage 0 breast cancer according to the TNM classification (UICC 8th edition). Given the presence of nipple retraction and the inability to exclude tumor involvement of the nipple, nipple preservation was considered unfeasible. Based on the MRI findings, partial excision including the nipple was deemed appropriate, and the patient was informed that delayed nipple reconstruction could be performed if postoperative cosmetic outcomes were unsatisfactory. However, she ultimately elected to undergo total mastectomy. The postoperative course was uneventful, and the patient was discharged in good condition.

**Fig. 2:**
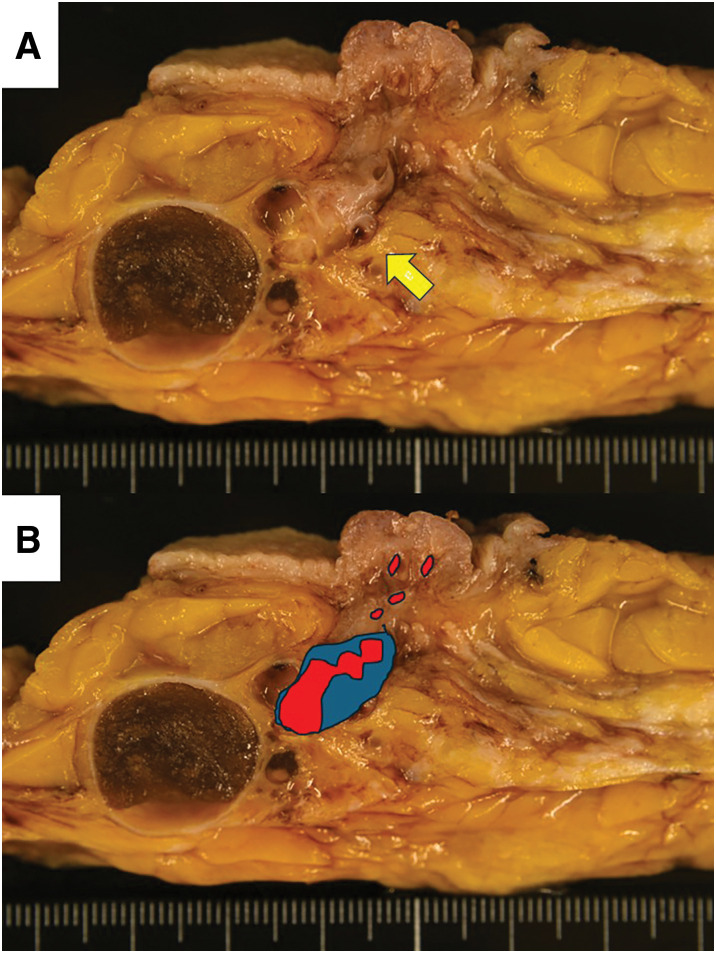
A sectional view demonstrates the relationship between the cyst, the tumor, and the nipple. (**A**) The cut surface shows a well-circumscribed, white solid tumor (arrow). (**B**) A diagram illustrating the relationships among PA, cysts, DCIS, and the nipple. The tumor is composed of the apocrine DCIS (red areas) and PA (blue area). DCIS, ductal carcinoma *in situ*; PA, pleomorphic adenoma

Macroscopically, the left total mastectomy specimen measured 22.0 × 19.0 cm and weighed 471 g. The sectioning of the specimen revealed a small breast mass near multiple cysts. It was well circumscribed, white, solid, and measured 1.3 × 1.0 cm without necrosis (**Fig. 2**). No extension into the skin of the nipple or fatty tissue was observed.

**Fig. 3:**
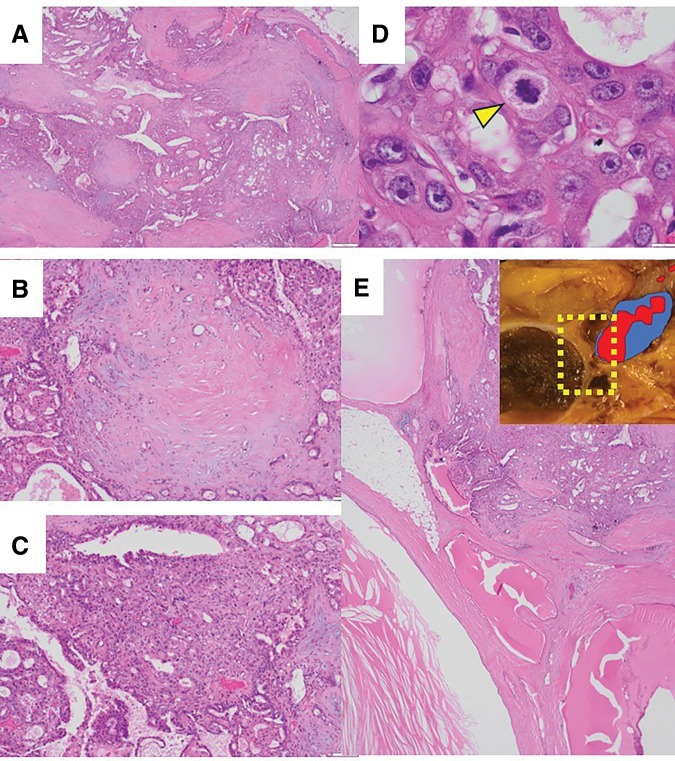
Microscopic features of apocrine DCIS ex PA on the total mastectomy specimen. No necrotic areas are identified within the tumor. (**A**) The stroma shows a mixture of myxomatous and hyalinized fibrous components (hematoxylin–eosin, original magnification ×20). (**B**) The tumor consists of PA with myxomatous stroma (hematoxylin–eosin, original magnification ×100). (**C**) A focus of apocrine DCIS is observed contiguous with the PA (hematoxylin–eosin, original magnification ×100). (**D**) The apocrine DCIS cells exhibit abundant eosinophilic cytoplasm, prominent nucleoli, and a few mitotic figures (arrowhead) (hematoxylin–eosin, original magnification ×1000). (**E**) The cyst and the pleomorphic adenoma were separate, with no continuity between them. DCIS, ductal carcinoma *in situ*; PA, pleomorphic adenoma

**Fig. 4:**
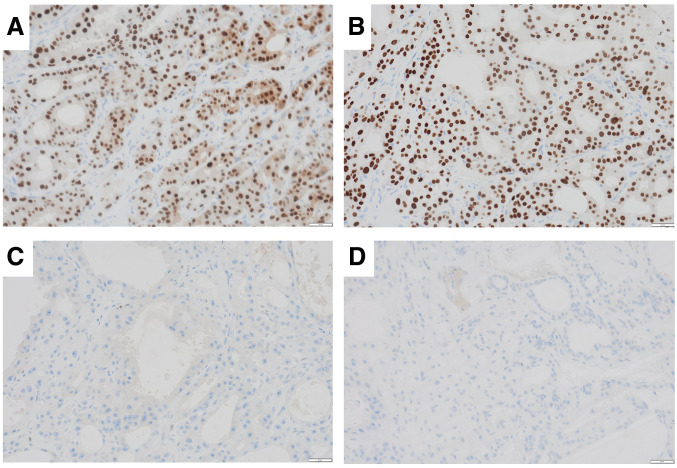
Immunophenotypic features of apocrine DCIS. The tumor is positive for AR (**A**) and FOXA1 (**B**) but negative for ER (**C**) and PgR (**D**). AR, androgen receptor; DCIS, ductal carcinoma *in situ*; ER, estrogen receptor; FOXA1, forkhead box protein A1; PgR, progesterone receptor

Microscopically, the tumor appeared as a well-defined, encapsulated neoplasm with a myxomatous stroma or hyalinized fibrous stroma (**Fig. 3A**). The tumor was composed of PA (**Fig. 3B**) and apocrine DCIS (**Fig. 3C**). DCIS cells had abundant eosinophilic cytoplasm. The nuclei were enlarged and exhibited round, oval, or pleomorphic shapes, with fine chromatin and a single inconspicuous nucleolus per nucleus (**Fig. 3D**); they were of high nuclear grade. A few mitotic activities were observed. The cystic lesion and the tumor on the nipple side were separate, with no continuity observed between them (**Fig. 3E**). The tumor was composed of the apocrine DCIS and PA. Cancer cells had spread within the ducts up to near the nipple, which was considered the cause of the nipple retraction; however, no invasion beyond the ducts was observed (**Fig. 2B**). Immunohistochemically, tumor cells tested positive for AR (**Fig. 4A**) and FOXA1 (**Fig. 4B**). Still, they were negative for ER (**Fig. 4C**) and PgR (**Fig. 4D**). The Ki-67 labeling index was 30%.

## DISCUSSION

PA is a benign tumor that is commonly found in the salivary glands. PA of the breast was initially described by Smith and Taylor in 1969.^1)^ PA probably starts as an intraductal papilloma.^2)^ The myoepithelial cells of the papilloma are extraordinarily stimulated, resulting in the formation of the characteristic stromal elements.^3)^ Breast PA usually locates around the subareolar area, with a wide age range.^4)^ Reports of PA in the breast are extremely rare, and Ahmad et al. conducted a review of 77 cases in 2023.^5)^ There are few physical examination findings or imaging findings that distinguish malignant tumors from PA. Management of PA of the breast involves complete surgical excision with sufficient margins. Although it is a benign neoplasm, an inadequate excision can result in recurrence, and even multiple recurrences have been described. In addition, numerous recurrences are prone to malignant transformation.

CEPA is a malignant tumor that arises from a pre-existing PA. It represents the malignant transformation of a previously benign lesion. CEPA is most commonly observed in the salivary glands, but cases involving the mammary glands, lacrimal glands, trachea, and nasal cavity have also been reported.^6)^ Malignant change in the mammary glands is very rare, where only 6 cases have been reported in 3 reports.^7–9)^ Our case represents the seventh reported instance of malignant transformation of PA in the breast and the first case associated with apocrine DCIS ex PA.

Breast cysts are thought to result mainly from ductal dilatation and the accumulation of secretions due to ductal obstruction. PA is thought to originate from an intraductal adenoma, which is an intraductal lesion. It is well established that an intraductal papilloma may obstruct the duct in which it arises, and the accumulation of fluid produced by the lesion subsequently results in cyst formation. In this case, a PA arose from a pre-existing intraductal papilloma, and DCIS subsequently developed within the PA and extended toward the nipple. The presence of the intraductal papilloma and PA was presumed to have increased the intraductal pressure, and the development of DCIS in the same region may have further elevated the pressure within the ductal system. The cysts located on the peripheral side of the PA were considered to have formed in response to this rise in intraductal pressure, and the nipple inversion was thought to have been caused by tissue changes occurring directly beneath the nipple. The precise etiology of breast cysts remains incompletely understood; however, hormonal fluctuations and the normal processes of breast development and involution have been implicated in their formation. In addition, ductal obstruction resulting in ductal dilatation and intraductal fluid retention has been proposed as a contributing mechanism, as observed in conditions such as galactocele.^10)^

## CONCLUSIONS

CEPA in the breast is exceptionally uncommon, and this report describes the first case of apocrine DCIS arising from PA. In this patient, ductal obstruction by the PA and associated DCIS likely caused peripheral cyst formation and nipple retraction. Awareness of this rare entity is essential for accurate diagnosis and optimal surgical management.

## DECLARATIONS

### Availability of data and materials

All data generated or analyzed during this study are included in this article and/or its supplementary material files. Further inquiries can be directed to the corresponding author.
